# Supplementary material for: A high-resolution temporal atlas of the SARS-CoV-2 translatome and transcriptome
Source: Nat Commun. 2021 Aug 25;12:5120. doi: 10.1038/s41467-021-25361-5 (PMC8387416; doi:10.1038/s41467-021-25361-5)
Supplement: Supplementary file 1 — Supplementary Information [file 41467_2021_25361_MOESM1_ESM.pdf]

Supplementary Information

A high-resolution temporal atlas of the SARS-CoV-2 translatome and transcriptome

Doyeon Kim, Sukjun Kim, Joori Park, Hee Ryung Chang, Jeeyoon Chang, Junhak Ahn, Heedo Park, Junehee Park, Narae Son, Gihyeon Kang, Jeonghun Kim, Kisoon Kim, Man-Seong Park, Yoon Ki Kim, Daehyun Baek

Supplementary Figures ········ 2

Supplementary Tables ········ 24

Supplementary Figures

Supplementary Figure 1

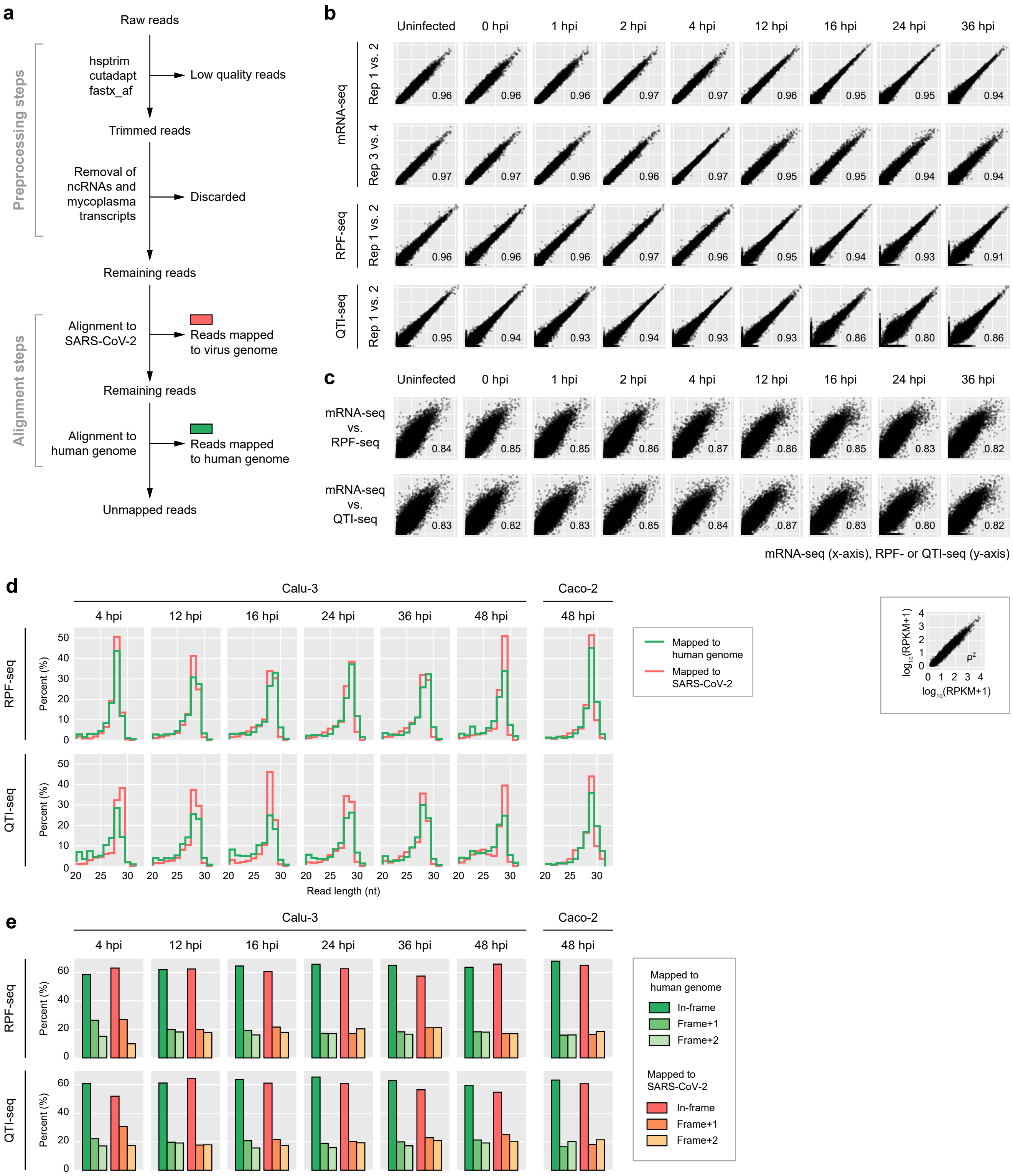

Supplementary Figure 1. Experimental design and generation of massive-scale datasets.

a, Details of mRNA-seq, RPF-seq, QTI-seq, and sRNA-seq data preprocessing and read alignment. After filtering out the low-quality reads and the reads mapped on ncRNAs such as tRNAs and rRNAs, the reads were first aligned to the SARS-CoV-2 genome. The remaining reads were then aligned to the human genome.

b, Full version for high reproducibility between the replicates of our sequencing data. Otherwise as in **Fig. 1b**.

c, High reproducibility between mRNA-seq and RPF-seq data. For each hpi, Spearman's ρ was calculated by comparing mRNA-level and RPF-level gene expression (left). The x-axis indicates mRNA-seq $\log_{10}$(RPKM+1) averaged between the replicates, and the y-axis indicates averaged RPF-seq $\log_{10}$(RPKM+1). A similar analysis was repeated by comparing mRNA-seq and QTI-seq (right). Otherwise as in (**b**).

d, Distribution of read lengths for translatome dataset. For RPF-seq (top) and QTI-seq (bottom) reads mapped to human (green) or SARS-CoV-2 genome (red), distributions of the read lengths are shown for each time point and each cell line.

e, Triplet nucleotide periodicity of translatome dataset. The 13th nucleotide position (12-nt offset from the 5′ end) of the reads mapped to human (green) or SARS-CoV-2 (red), indicating the ribosome P-site position, was counted for RPF-seq (top) and QTI-seq (bottom). The relative fractions of the ribosome P-sites mapped to each of three codon nucleotides are shown for each time point and each cell line. Open reading frames are depicted as three different colored bars with the darkest bars indicating in-frame and the others out-of-frames.

Supplementary Figure 2

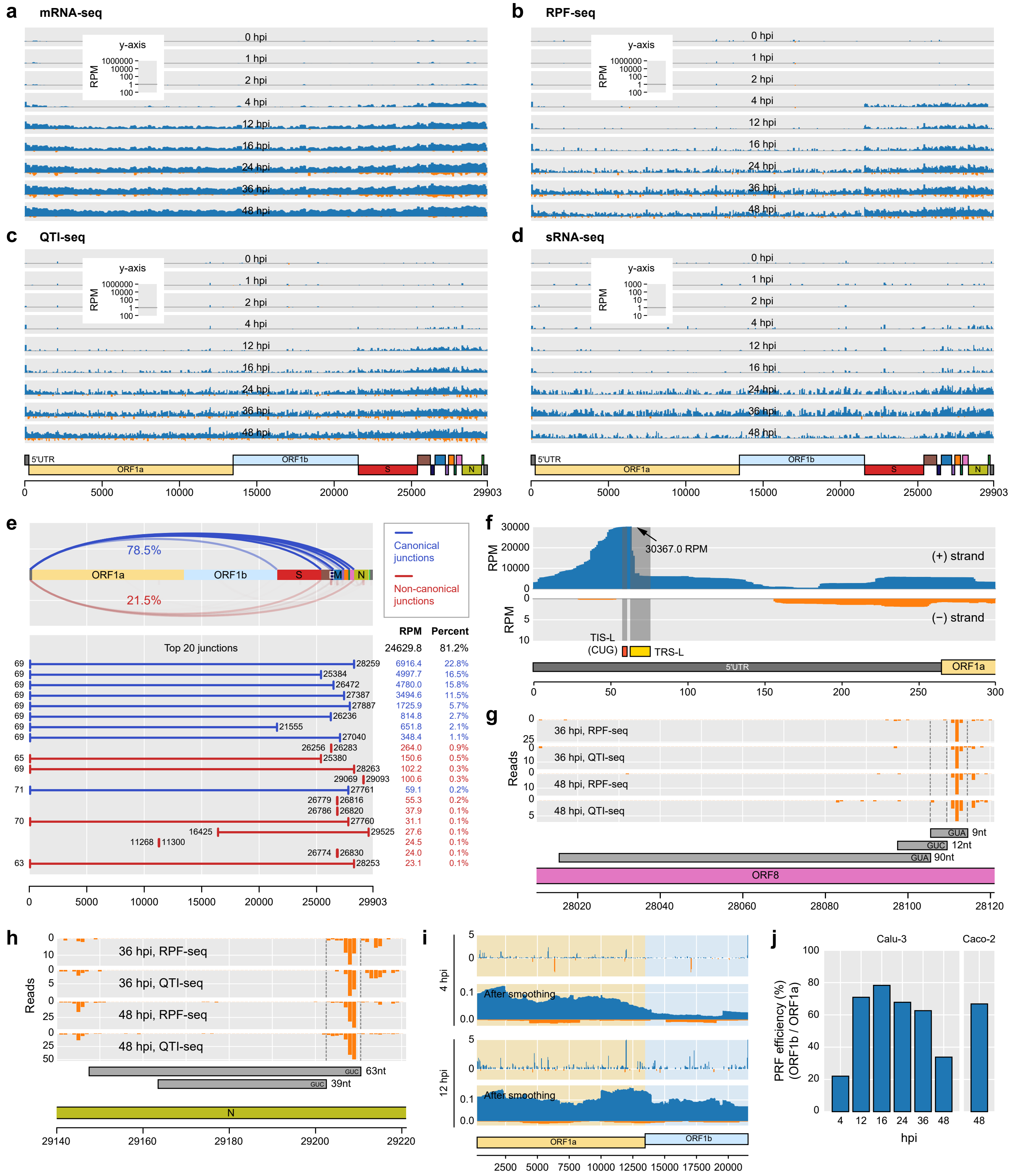

Supplementary Figure 2. Temporal landscape of the SARS-CoV-2 translatome and transcriptome.

a-d, A full version for coverage of mRNA-seq (**a**), RPF-seq (**b**), QTI-seq (**c**), and sRNA-seq (**d**) reads across the SARS-CoV-2 genome from early to late phase (0 to 36 hours post-infection (hpi)) after viral infection (multiplicity of infection (MOI)=10), and at 48 hpi (MOI=0.1). Otherwise as in **Fig. 2a**.

e, Canonical and non-canonical junctions observed in discontinuous transcription. Junctions mediated by transcriptional regulatory sequences (TRS-L and TRS-B) are annotated as 'canonical' and the others as 'non-canonical'. Frequently detected top 20 junctions were displayed at the bottom. The number of reads per million mapped reads (RPM) was calculated for each junction, and relative fractions of junctions detected in all mRNA-seq samples at 36 hpi were presented.

f, Read coverage plots of mRNA-seq for 5′ end (position 0 – 300) of the SARS-CoV-2 genome at 36 hpi. The read depths of positive and negative strands were presented in blue and orange, respectively, on a natural scale of RPM. Translation initiation site located in 5′ leader (TIS-L) and TRS-L are depicted as orange and yellow boxes, respectively.

g, h, Enrichment of RPF-seq and QTI-seq reads mapped to negative strand of the SARS-CoV-2 genome. The positions where the maximum sum of RPF-seq and QTI-seq reads were mapped at 36 hpi (28012th nt position; **g**) and 48 hpi (29209th nt position; **h**) are displayed. Predicted ORFs that initiate from an AUG/CUG codon located near the position with the maximum sum of RPF-seq and QTI-seq reads are depicted as grey horizontal bars with their start codons and the predicted ORF sizes shown. Otherwise as in **Fig. 3b**.

i, Read coverages of ORFs 1a and 1b in RPF-seq samples at 4 hpi and 12 hpi. Read coverage at a nucleotide resolution (the first row) and smoothed read coverage (the second row) with moving average of a 4-kb sliding window are shown. The x-axis represents the nucleotide position of the SARS-CoV-2 genome.

j, Efficiency of programmed ribosomal frameshifting (PRF) between ORF1a and ORF1b measured for each time point and each cell line.

Supplementary Figure 3

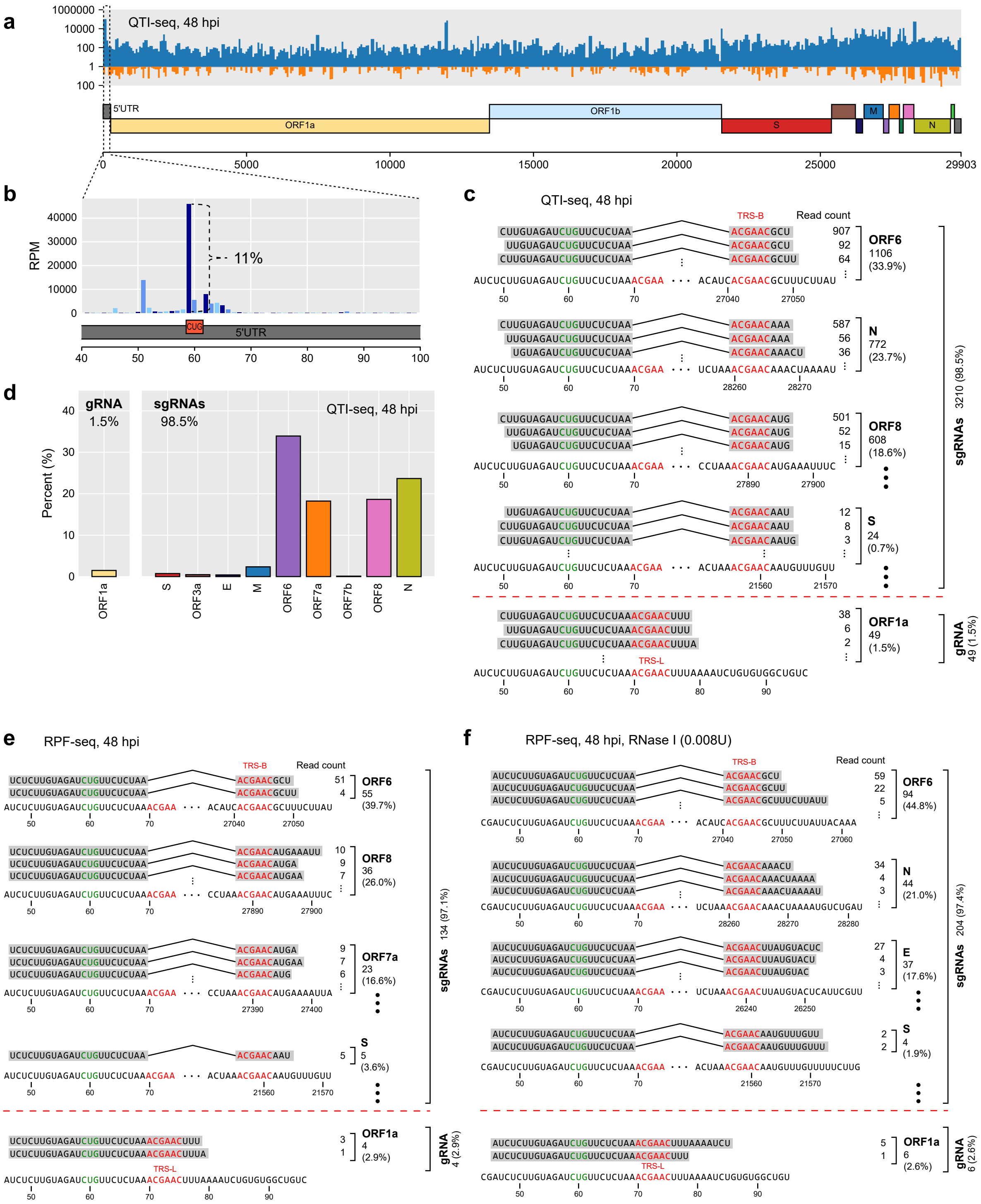

Supplementary Figure 3. Extensive translation initiation by a translation initiation site located in the leader (TIS-L) for both gRNA and sgRNAs (QTI-seq).

a, Coverage of QTI-seq reads across the SARS-CoV-2 genome at 48 hours post-infection (hpi) (multiplicity of infection=0.1). Otherwise as in **Fig. 3a**.

b, Enrichment of QTI-seq reads at the TIS-L. Otherwise as in **Fig. 3b**.

c, 3′ ends of QTI-seq reads mapped on TIS-L at 48 hpi. Otherwise as in **Fig. 3c**.

d, The relative fraction of QTI-seq TIS-L reads uniquely mapped to each ORF calculated in (**c**). Otherwise as in **Fig. 3d**.

e, f, 3′ ends of RPF-seq reads mapped on TIS-L with a subset of RPF-seq reads more confidently mapped to TIS-L. For 48 hpi (**e**), a subset of long RPF-seq reads mapped to C nucleotide position of TIS-L were collected (**Methods**). The alignments of those uniquely mapped reads to the gRNA or sgRNAs are displayed and the corresponding read counts with the relative fraction of reads mapped to each ORF are shown in parentheses. Similar analysis was performed for RPF-seq dataset with low RNase I concentration (**f**). Otherwise as in **Fig. 3c, e**.

Supplementary Figure 4

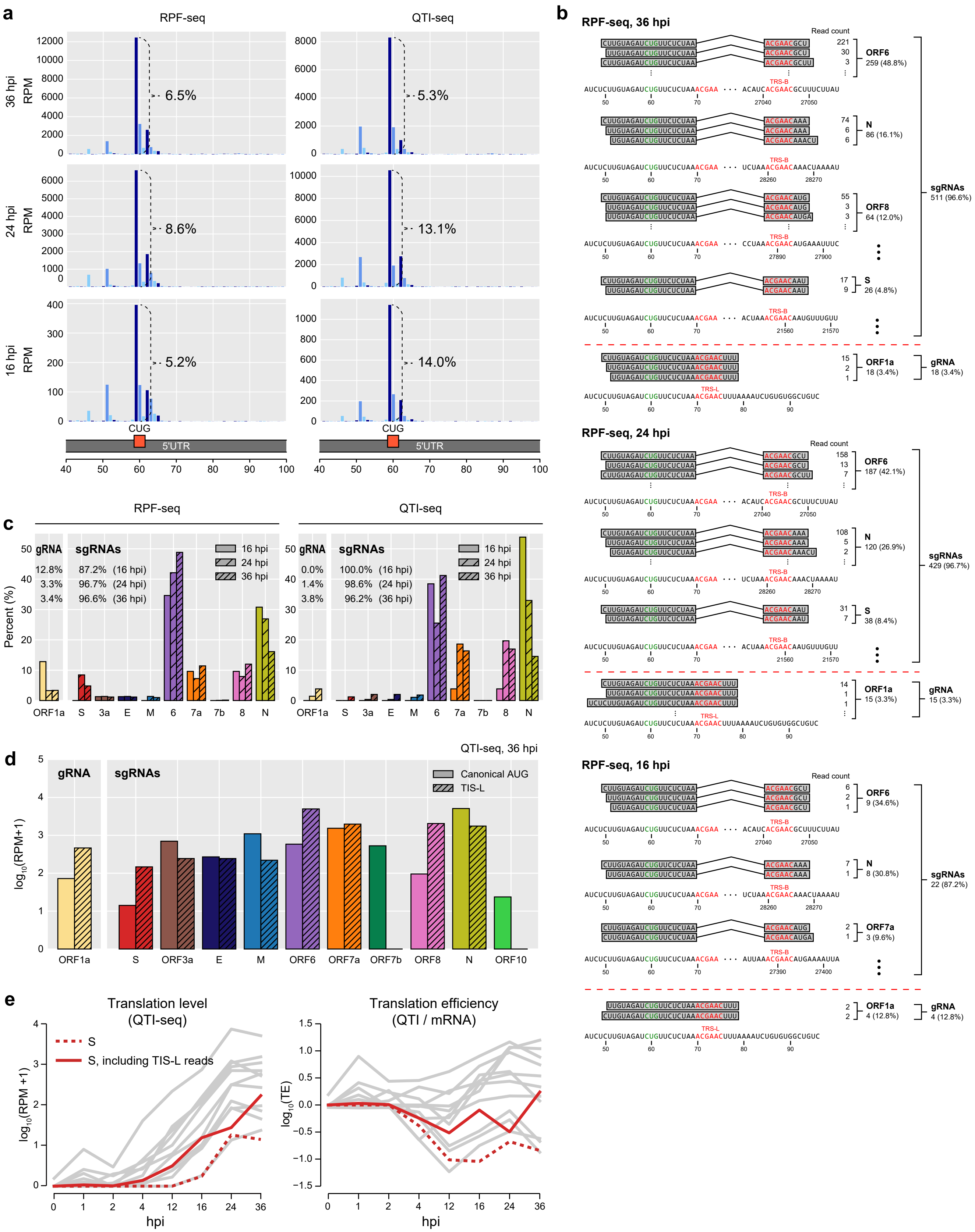

Supplementary Figure 4. Evidence for extensive translation initiation by a translation initiation site located in the leader (TIS-L) for both gRNA and sgRNAs.

a, Enrichment of RPF-seq (left) and QTI-seq (right) reads mapped on TIS-L at 36 (top), 24 (middle), and 16 (bottom) hours post-infection (hpi) (multiplicity of infection=10). Otherwise as in **Fig. 3b**.

b, 3′ ends of RPF-seq reads uniquely mapped on TIS-L for Calu-3 cells at 36 (top), 24 (middle), and 16 (bottom) hpi. Otherwise as in **Fig. 3c**.

c, The relative fraction of RPF-seq (left) and QTI-seq (right) TIS-L reads uniquely mapped to each ORF at 16, 24, and 36 hpi. Otherwise as in **Fig. 3d**.

d, For each ORF, the level of translation initiation at TIS-L is compared with that at the annotated translation initiation site using QTI-seq dataset for 36 hpi. Otherwise as in **Fig. 3f**.

e, Translation level (left) and translation efficiency (right) of ORF S observed in QTI-seq datasets. Otherwise as in **Fig. 3g**.

Supplementary Figure 5

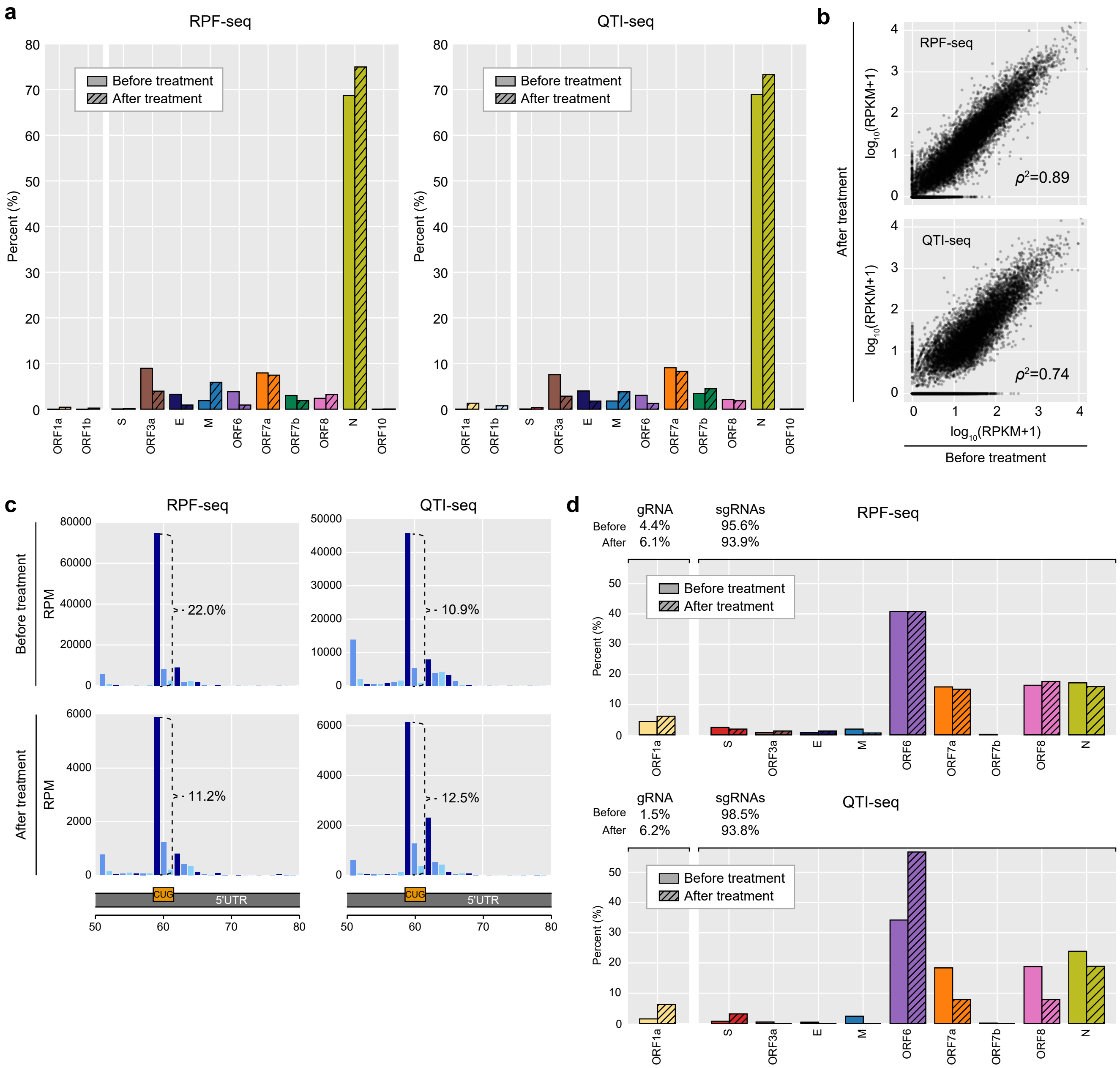

Supplementary Figure 5. Comparison of translatome datasets before and after mycoplasma contamination.

a, Comparison of the RPF-seq (left) and QTI-seq (right) expression levels of SARS-CoV-2 ORFs before and after treatment in Calu-3 cell lines at 48 hours post-infection (hpi). The relative fraction of the expression level of each ORF was represented as a percentage.

b, Comparison of the RPF-seq (top) and QTI-seq (bottom) expression levels of human protein-coding genes before and after treatment in Calu-3 cell lines at 48 hpi. Correlation coefficient (Spearman's ρ) was calculated by comparing host gene expression levels between translatome samples before and after treatment for mycoplasma contamination. Both x- and y-axes represent $\log_{10}$(RPKM+1).

c, Enrichment of RPF-seq (left) and QTI-seq (right) reads on the translation initiation site located in the leader (TIS-L) for Calu-3 cell line before treatment for mycoplasma infection (top) and after treatment (bottom). The 13th position (12-nt offset from the 5′ end) of the reads, indicating the ribosome P-site position, was counted and calculated as the number of reads per million mapped reads (RPM). Open reading frames are represented by three different colored bars with the dark blue bars indicating in-frame with TIS-L and the others out-of-frames.

d, Comparison of the relative fractions of RPF-seq (left) and QTI-seq (right) TIS-L reads uniquely mapped to each of gRNA and sgRNAs for Calu-3 cell line before treatment for mycoplasma infection and after treatment.

Supplementary Figure 6

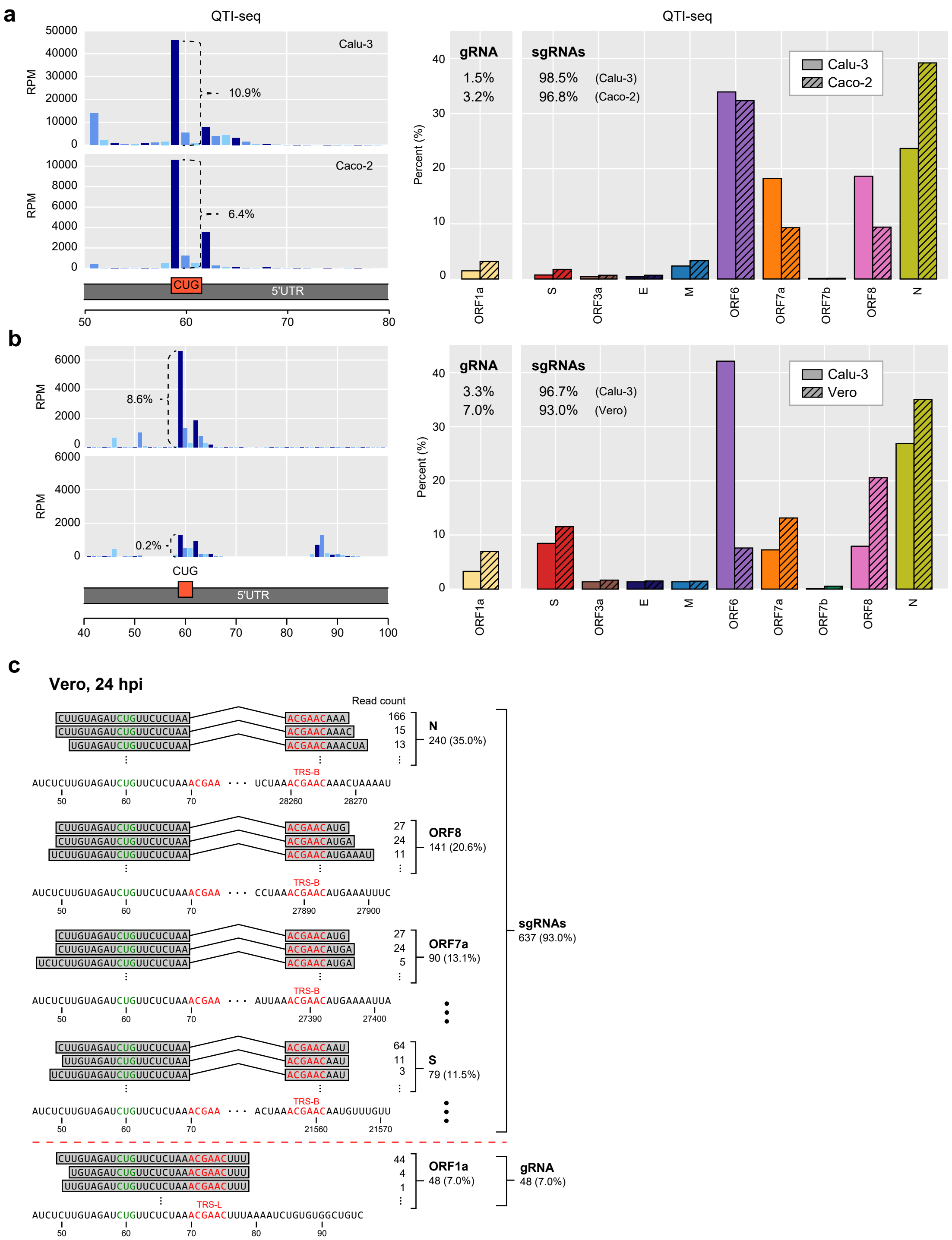

Supplementary Figure 6. Translation initiation site located in the leader (TIS-L) is active in multiple cell lines.

a, Enrichment of QTI-seq reads at the TIS-L for Calu-3 and Caco-2 cell lines at 48 hours post-infection (hpi) (left). The relative fractions of TIS-L reads mapped to each of gRNA and sgRNAs are compared between Calu-3 and Caco-2 (right). Otherwise as in **Fig. 3h**.

b, Enrichment of RPF-seq reads at the TIS-L for Calu-3 and Vero cell lines at 24 hpi (left). The relative fractions of TIS-L reads mapped to each of gRNA and sgRNAs are compared between Calu-3 and Vero (right). Otherwise as in **Fig. 3h**.

c, 3′ ends of RPF-seq reads uniquely mapped on TIS-L for Vero cells at 24 hpi. Otherwise as in **Fig. 3c**.

Supplementary Figure 7

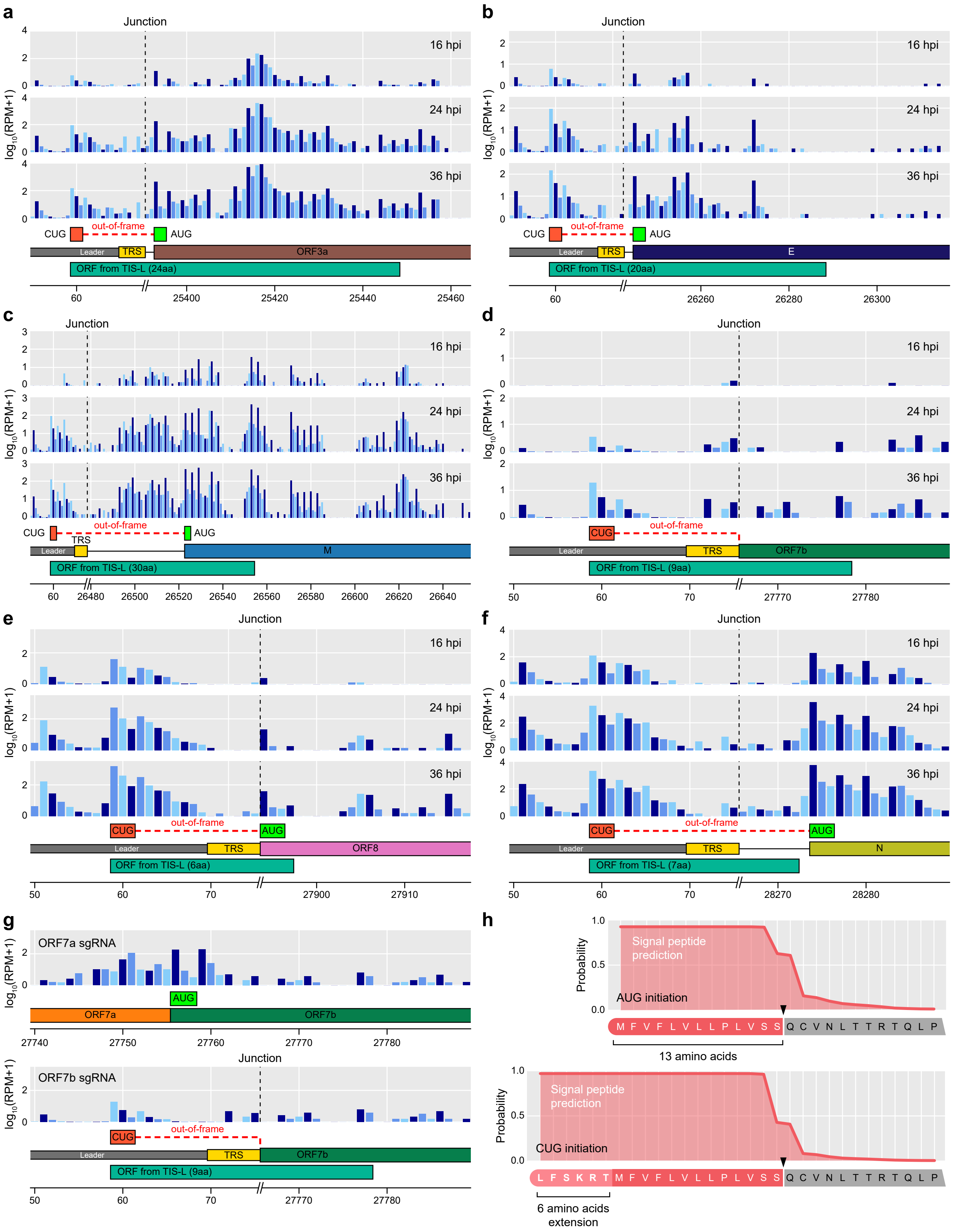

Supplementary Figure 7. Translation initiation site located in the leader (TIS-L) functions as a global regulator of the SARS-CoV-2 translatome (RPF-seq).

a-f, RPF-seq reads mapped on TIS-L compared to those of the annotated ORFs 3a (**a**), E (**b**), M (**c**), 7b (**d**), 8 (**e**), and N (**f**), measured at 16, 24, and 36 hours post-infection (hpi) with their nucleotide sequence, annotation of each ORF, TRS, TIS-L, and a predicted ORF initiated from TIS-L shown below. Otherwise as in **Fig. 4**.

g, Translation of ORF 7b is likely to be occurred by leaky scanning of ORF 7a sgRNA. RPF-seq reads around the start codon of ORF 7b in ORF 7a sgRNA (top) were compared to those of ORF 7b sgRNA (bottom) at 36 hpi. Otherwise as in **Fig. 4**.

h, Predicted signal peptide and its cleavage site of the spike protein. The signal peptide and its cleavage site of the spike protein assuming translation is either initiated from AUG (top) or CUG (bottom) were predicted using SignalP-5.0. The y-axis represents the probability of the site being the signal peptide. The predicted cleavage sites of the signal peptide are depicted as black triangles.

Supplementary Figure 8

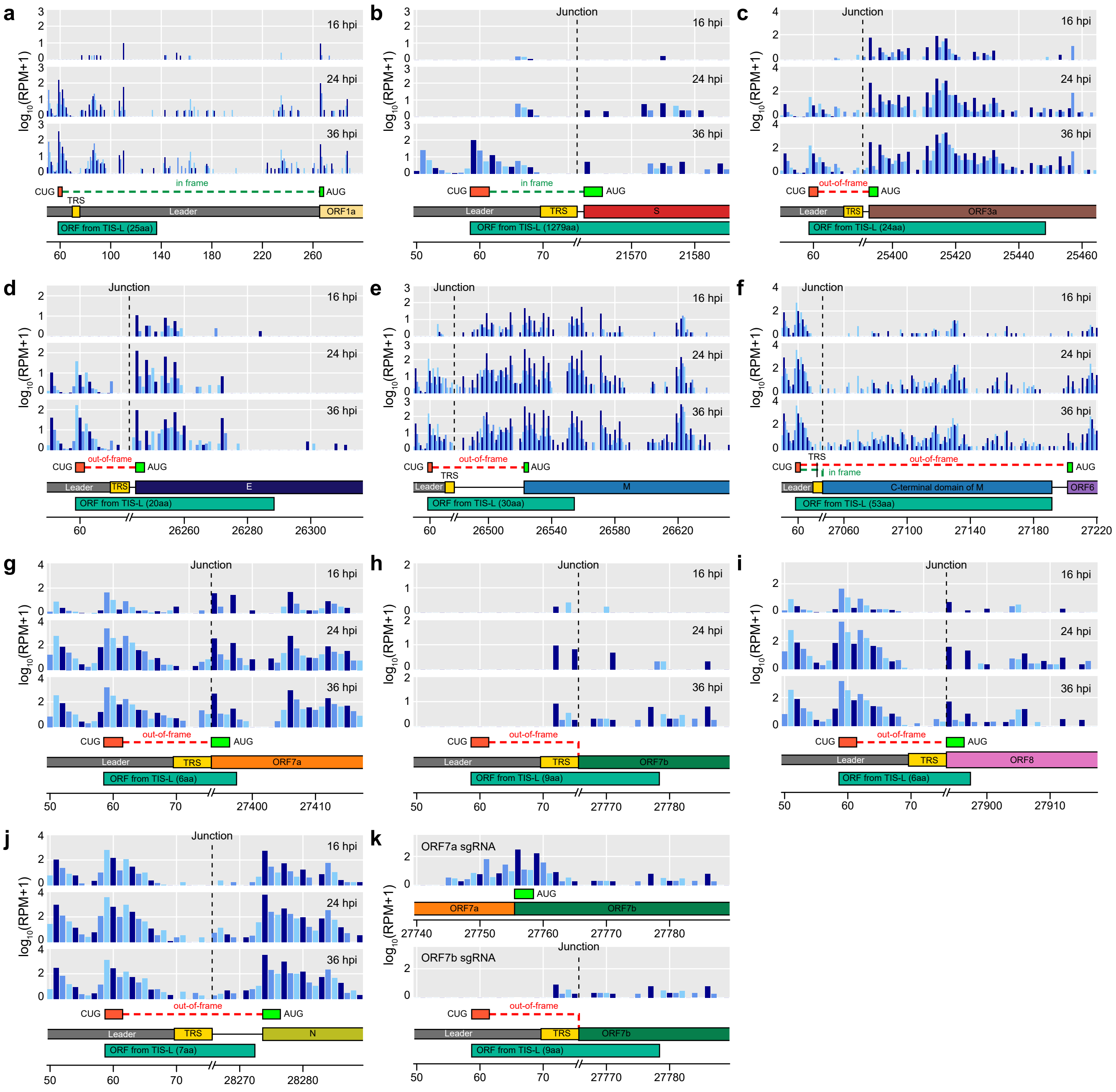

Supplementary Figure 8. Translation initiation site located in the leader (TIS-L) functions as a global regulator of the SARS-CoV-2 translatome (QTI-seq and validation experiment).

a-j, QTI-seq reads mapped on TIS-L compared to those of the annotated regions of ORFs 1a (**a**), S (**b**), 3a (**c**), E (**d**), M (**e**), 6 (**f**), 7a (**g**), 7b (**h**), 8 (**i**), and N (**j**). Otherwise as in **Supplementary Fig. 7**.

k, Translation of ORF 7b by leaky scanning of ORF 7a sgRNA observed in QTI-seq. Otherwise as in **Supplementary Fig. 7g**.

Supplementary Figure 9

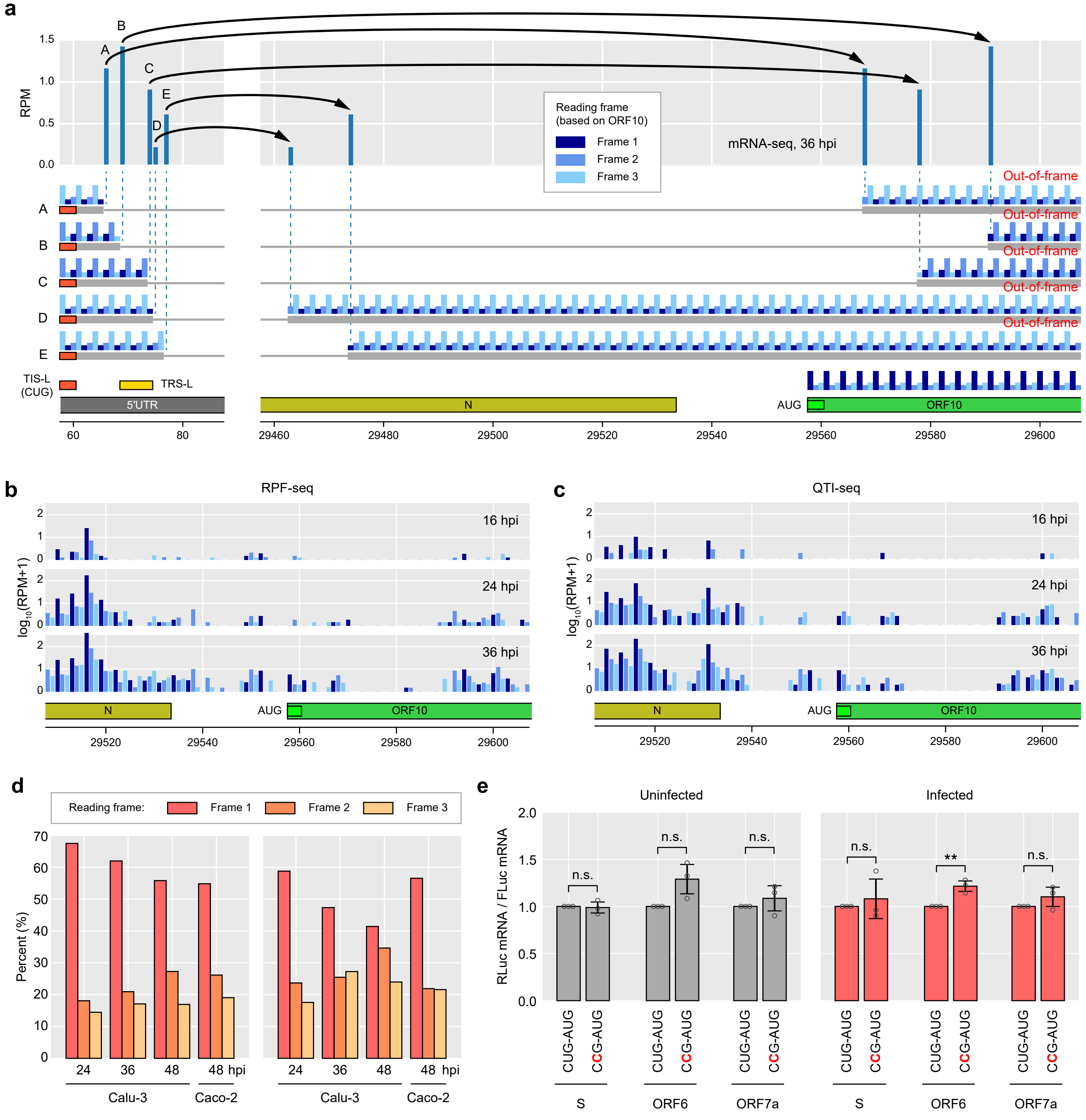

Supplementary Figure 9. Transcription and translation of ORF10 and experimental validation of translation initiation site located in the leader (TIS-L).

a, Non-canonical ORF 10 sgRNAs. From mRNA-seq at 36 hours post-infection (hpi), the reads that include a junction located in the region between 100 nt upstream and 50 nt downstream of ORF 10 start position were counted and calculated as RPM. The most abundant five junction pairs are shown with their genomic positions. ORF 10 sgRNAs created from each junction and their reading frames to ORF 10 are depicted below. Open reading frames are depicted as three different colored bars with the darkest bars indicating in-frame and the others out-of-frames with respect to ORF 10.

b, Translation of ORF 10. RPF-seq reads around the start codon of ORF 10 were visualized. Otherwise as in **Fig. 4**.

c, Translation of ORF 10 observed in QTI-seq. Otherwise as in (**b**).

d, Triplet nucleotide periodicity of the reads mapped to ORF 10. Otherwise as in **Supplementary Fig. 1e**.

e, Relative levels of RLuc mRNAs in **Fig. 4f**. The levels of RLuc mRNA were normalized to those of FLuc mRNA (Two-tailed, equal-sample variance Student's *t*-tests, *P<0.05, **P<0.01, ***P<0.001). The mean values ±s.d. are displayed (n=3 biologically independent experiments). *P* values are provided in Source Data.

Supplementary Figure 10

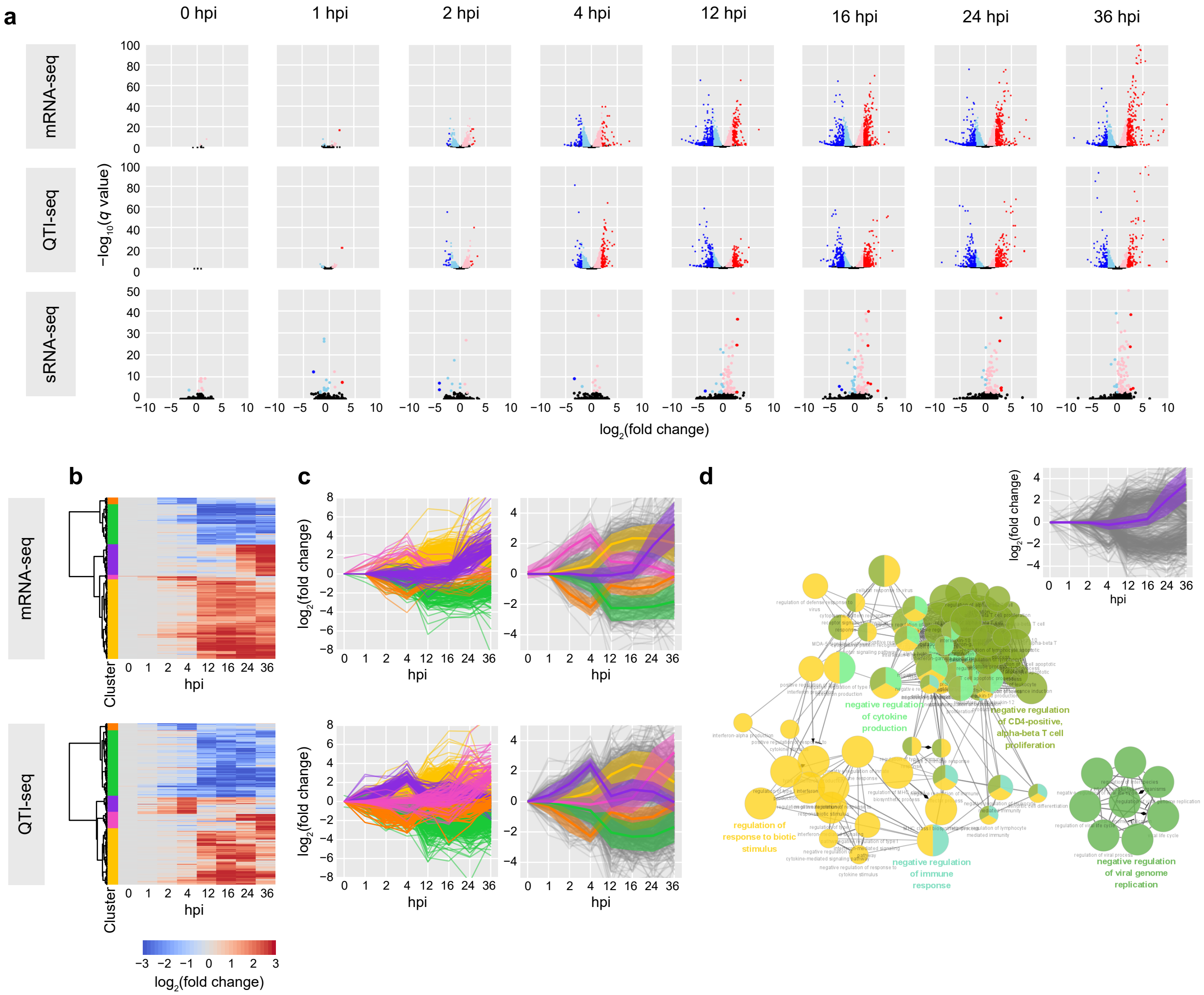

Supplementary Figure 10. Early and late responding human genes to SARS-CoV-2 infection.

a, Differentially expressed genes (DEGs) identified from mRNA-seq (top), QTI-seq (middle), and sRNA-seq (bottom). Otherwise as in **Fig. 5a**.

b, Hierarchical clustering of the DEGs displayed in mRNA-level (top) and QTI-level (bottom). Otherwise as in **Fig. 5b**.

c, Temporal expression changes of the identified DEGs in mRNA-level (top) and QTI-level (bottom), color-coded for the five clusters determined in (**b**). Otherwise as in **Fig. 5c**.

d, Gene Ontology enrichment analysis for a cluster of DEGs in RPF-level determined in **Fig. 5b**, but not displayed in **Fig. 5d-g**. Otherwise as in **Fig. 5d**.

Supplementary Figure 11

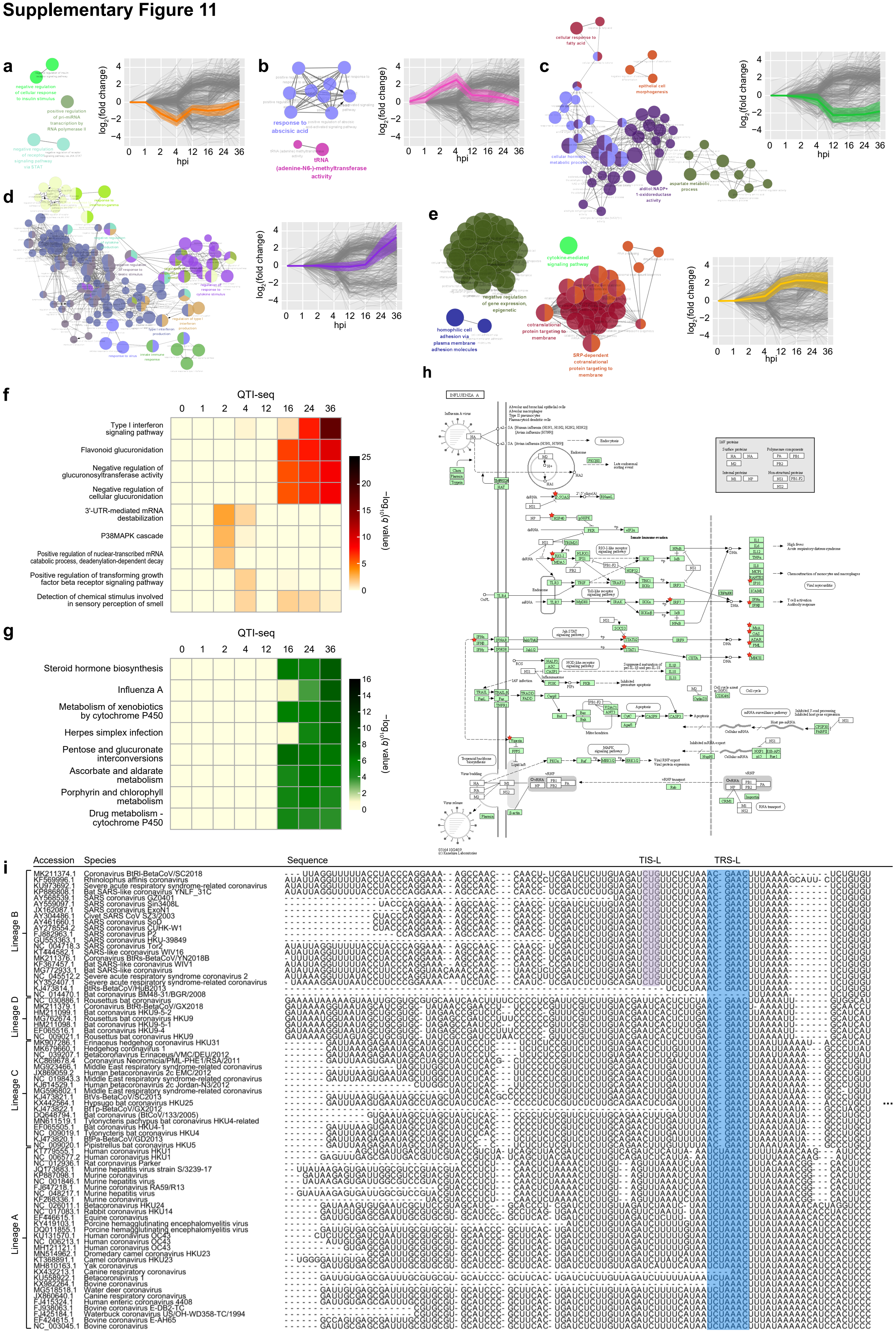

Supplementary Figure 11. Associated functions and pathways of human genes responding to SARS-CoV-2 infection.

a-e, Gene Ontology (GO) enrichment analysis for a cluster of differentially expressed genes (DEGs) in mRNA-level, determined by analysis of **Supplementary Fig. 10b**. Otherwise as in **Fig. 5d**.

f, GO terms associated with DEGs identified from QTI-seq. Otherwise as in **Fig. 6f**.

g, DAVID KEGG pathways associated with DEGs identified from QTI-seq. Otherwise as in **Fig. 6g**.

h, DAVID KEGG pathway diagram for the influenza A pathway associated with DEGs (red star) identified from RPF-seq data at 36 hours post-infection. Gene products are shown as boxes, chemical compounds, DNA, and other molecules as circles. Molecular interactions, relations, and links are shown as arrows. Human specific pathways are displayed green.

i, Multiple sequence alignments for the 5′ leader region of various betacoronaviruses that belong to lineages A, B, C, and D.

Supplementary Tables

Supplementary Table 1. The number of reads at pre-processing step.

The number of replicates, sequenced reads, and discarded reads at pre-processing steps are described for sequencing datasets. The numbers of discarded reads at FASTQ quality trimming (step 1), adapter trimming (step 2), and artifact removal step (step 3) are shown. The number of reads mapped to non-coding transcripts including tRNAs, rRNAs, and other non-coding RNAs is shown in the column 'step 4'. The number of reads mapped to the mycoplasma genomes and thus discarded is listed in the column 'step 5'. The percentages in the parentheses were calculated by dividing the number of reads by the total sequenced reads. The number of remaining reads after 5 pre-processing steps is shown in the column 'remaining'. These reads were used for alignment and further analyses.

Calu-3 samples

assay	infection	replicates	sequenced	step 1	step 2	step 3	step 4	step 5	remaining
sRNA-seq	Uninfected	2	45,026,294	1,936,117	13,754,089	261	18,763,999 (41.7%)	800,423 (1.8%)	9,771,405
sRNA-seq	0 hpi	1	22,311,040	797,096	8,853,549	175	10,844,041 (48.6%)	511,359 (2.3%)	1,304,820
sRNA-seq	1 hpi	1	21,665,030	790,584	8,999,321	120	10,379,598 (47.9%)	548,268 (2.5%)	947,139
sRNA-seq	2 hpi	2	53,798,253	1,575,552	16,117,463	701	30,957,957 (57.5%)	2,097,808 (3.9%)	3,048,772
sRNA-seq	4 hpi	2	55,796,404	1,726,186	17,476,148	540	30,557,112 (54.8%)	2,965,013 (5.3%)	3,071,405
sRNA-seq	12 hpi	1	17,819,476	565,180	1,492,912	55	12,049,831 (67.6%)	319,114 (1.8%)	3,392,384
sRNA-seq	16 hpi	1	25,740,487	629,746	2,085,204	89	15,845,985 (61.6%)	279,155 (1.1%)	6,900,308
sRNA-seq	24 hpi	1	10,475,478	260,339	605,435	51	6,926,281 (66.1%)	162,817 (1.6%)	2,520,555
sRNA-seq	36 hpi	1	24,883,176	674,072	2,950,028	87	14,128,613 (56.8%)	276,598 (1.1%)	6,853,778
sRNA-seq	48 hpi	1	26,820,359	1,337,294	7,645,836	58	5,869,397 (21.9%)	259,232 (1.0%)	11,708,542
mRNA-seq	Uninfected	10	288,542,388	10,482,05 2	2,566,896	6,032	122,286,109 42.4%	49,169,090 (17.0%)	104,032,209
mRNA-seq	0 hpi	4	109,615,974	3,670,486	490,425	3,074	39,238,160 35.8%	18,962,281 (17.3%)	47,251,548
mRNA-seq	1 hpi	4	119,225,613	3,745,977	636,468	2,797	46,524,335 (39.0%)	19,683,560 (16.5%)	48,632,476

mRNA-seq	2 hpi	4	107,587,508	3,381,038	612,091	2,803	44,741,859 (41.6%)	16,425,309 (15.3%)	42,424,408
mRNA-seq	4 hpi	4	104,210,824	3,217,492	1,413,919	2,703	49,416,613 (47.4%)	12,569,073 (12.1%)	37,591,024
mRNA-seq	12 hpi	4	80,172,520	2,667,146	3,790,112	1,286	33,589,630 (41.9%)	11,096,699 (13.8%)	29,027,647
mRNA-seq	16 hpi	4	81,862,979	2,903,917	5,263,966	1,137	40,729,328 (49.8%)	11,155,841 (13.6%)	21,808,790
mRNA-seq	24 hpi	4	76,850,194	2,481,836	2,331,072	847	35,896,365 (46.7%)	12,553,905 (16.3%)	23,586,169
mRNA-seq	36 hpi	4	77,368,984	2,477,257	1,704,673	890	34,494,591 (44.6%)	11,755,926 (15.2%)	26,935,647
mRNA-seq	48 hpi	6	170,199,865	6,635,884	1,559,173	1,853	61,466,652 (36.1%)	23,366,489 (13.7%)	77,169,814
RPF-seq	Uninfected	5	140,407,788	5,491,940	21,225,772	200	89,897,163 (64.0%)	3,879,844 (2.8%)	19,912,869
RPF-seq	0 hpi	2	54,423,427	1,458,940	6,774,540	83	37,411,067 (68.7%)	2,168,487 (4.0%)	6,610,310
RPF-seq	1 hpi	2	54,047,016	1,363,629	6,549,870	66	33,983,384 (62.9%)	1,953,449 (3.6%)	10,196,618
RPF-seq	2 hpi	2	54,548,807	1,351,618	6,192,193	56	33,048,976 (60.6%)	1,431,657 (2.6%)	12,524,307
RPF-seq	4 hpi	2	59,664,262	1,529,365	8,399,485	42	34,928,351 (58.5%)	1,747,511 (2.9%)	13,059,508
RPF-seq	12 hpi	2	46,750,727	1,322,140	1,190,058	14	39,779,904 (85.1%)	252,178 (0.5%)	4,206,433
RPF-seq	16 hpi	2	56,249,889	1,504,497	1,577,248	37	48,251,903 (85.8%)	729,631 (1.3%)	4,186,573
RPF-seq	24 hpi	2	47,938,414	1,296,573	1,309,253	19	42,101,925 (87.8%)	369,669 (0.8%)	2,860,975
RPF-seq	36 hpi	2	46,349,876	1,155,726	1,200,399	10	41,214,799 (88.9%)	493,105 (1.1%)	2,285,837
RPF-seq	48 hpi	3	77,010,524	3,753,426	14,421,763	139	51,102,057 (66.4%)	1,560,260 (2.0%)	6,172,879
QTI-seq	Uninfected	5	134,751,383	5,693,408	19,826,733	236	94,608,514 (70.2%)	3,043,977 (2.3%)	11,578,515
QTI-seq	0 hpi	2	55,679,206	1,671,563	6,101,827	110	42,197,987 (75.8%)	1,422,071 (2.6%)	4,285,648
QTI-seq	1 hpi	2	55,812,191	1,543,360	6,037,629	161	41,968,755 (75.2%)	1,320,501 (2.4%)	4,941,785
QTI-seq	2 hpi	2	61,231,630	1,733,330	6,280,581	99	45,436,578 (74.2%)	1,590,513 (2.6%)	6,190,529
QTI-seq	4 hpi	2	60,115,191	1,714,696	7,942,437	70	43,629,861 (72.6%)	1,373,425 (2.3%)	5,454,702
QTI-seq	12 hpi	2	49,678,760	1,436,592	1,419,982	14	43,562,377 (87.7%)	222,518 (0.4%)	3,037,277
QTI-seq	16 hpi	2	50,122,090	1,305,145	1,843,618	7	44,953,854 (89.7%)	251,565 (0.5%)	1,767,901
QTI-seq	24 hpi	2	44,003,422	1,085,428	1,253,684	20	40,207,605 (91.4%)	272,589 (0.6%)	1,184,096
QTI-seq	36 hpi	2	43,037,324	1,084,310	1,451,084	7	38,838,888 (90.2%)	241,690 (0.6%)	1,421,345
QTI-seq	48 hpi	3	69,420,456	3,976,950	12,005,006	141	48,050,772 (69.2%)	1,764,230 (2.5%)	3,623,357

Additional Calu-3 samples (After mycoplasma treatment)

assay	infection	replicates	sequenced	step 1	step 2	step 3	step 4	step 5	remaining
RPF-seq	48 hpi	2	25,973,973	2,876,155	1,193,554	6	18,112,172 (69.7%)	938 (0.0036%)	3,791,148
QTI-seq	48 hpi	2	23,394,787	2,547,160	1,599,246	6	13,964,075 (59.7%)	783 (0.0033%)	5,283,517

Caco-2 samples

assay	infection	replicates	sequenced	step1	step2	step3	step4	step5	remaining
RPF-seq	Uninfected	2	29,608,153	647,531	775,741	19	24,943,296 (84.2%)	833 (0.0028%)	3,240,733
RPF-seq	48 hpi	2	36,090,671	891,411	922,382	20	30,491,273 (84.5%)	5,556 (0.0154%)	3,780,029
QTI-seq	Uninfected	2	33,158,994	801,939	848,646	15	30,185,472 (91.0%)	287 (0.0009%)	1,322,635
QTI-seq	48 hpi	2	33,506,127	791,875	898,446	22	30,275,733 (90.4%)	3,948 (0.0118%)	1,536,103

Vero samples

assay	infection	replicates	sequenced	step1	step2	step3	step4	step5	remaining
RPF-seq	0 hpi	2	38,949,577	2,425,464	5,821,055	51	24,620,943 (63.1%)	1,287 (0.0033%)	6,080,777
RPF-seq	24 hpi	2	37,128,220	2,216,517	5,820,779	61	22,071,241 (59.4%)	1,730 (0.0047%)	7,017,892

Supplementary Table 2. List of primers used in the study

The primers used for qPCR and luciferase assays are listed. (FW: forward primer, RV: reverse primer)

Quantitative PCR

Name	Sequence (5′ to 3′)
FW: RLuc mRNA	TGATCCAGAACAAAGGAAAC
RV: RLuc mRNA	TAATACACCGCGCTACTGGCTCAA
FW: FLuc mRNA	CAACACCCCAACATCTTCG
RV: FLuc mRNA	CTTTCCGCCCTTCTTGGCC

Luciferase reporter assay

Name	Sequence (5′ to 3′)
FW: pCMV-S-C[C/U]G-RLuc	CTATATAAGCAGAGCTCGTTTAGTGAAC
RV: pCMV-S-C[C/U]G-RLuc	GATCATAAACTTTCGAAGTCATTGTTCG
FW: pCMV-ORF6-C[C/U]G-RLuc	CTATATAAGCAGAGCTCGTTTAGTGAAC
RV: pCMV-ORF6-C[C/U]G-RLuc	CTGGATCATAAACTTTCGAAGTCATCTG
FW: pCMV-ORF7a-C[C/U]G-RLuc	CTATATAAGCAGAGCTCGTTTAGTGAAC
RV: pCMV-ORF7a-C[C/U]G-RLuc	CATAAACTTTCGAAGTCATGTTCGTTTAG
