# Supplementary material for: A high-resolution temporal atlas of the SARS-CoV-2 translatome and transcriptome
Source: Nat Commun. 2021 Aug 25;12:5120. doi: 10.1038/s41467-021-25361-5 (PMC8387416; doi:10.1038/s41467-021-25361-5)
Supplement: Supplementary file 3 — Description of Additional Supplementary Information [file 41467_2021_25361_MOESM3_ESM.pdf]

Description of Additional Supplementary Files

File Name: Supplementary Data 1

Description: Hierarchical clustering of differentially expressed genes (DEGs). For mRNA-seq, RPF-seq, and QTI-seq, the DEGs with similar temporal expression patterns were grouped by hierarchical clustering. Lists of the DEGs are provided for each cluster.
